# Multi-Parameter Estimation for *κ*-*μ* Shadowed Fading Model in Wireless Sensor Networks

**DOI:** 10.3390/s26206520

**Published:** 2026-10-14

**Authors:** Liruizhi Xie, Bo Chen, Xianglu Li, Youyang Xiang, Qilong Du, Dong Hou, Jie Tian

**Affiliations:** 1 Institute of Electronic Engineering, China Academy of Engineering Physics, Mianyang 621900, China; xieliruizhi24@gscaep.ac.cn (L.X.); 202021220217@std.uestc.edu.cn (Q.D.); 2 School of Automation Engineering, University of Electronic Science and Technology of China, Chengdu 611731, China; houdong@uestc.edu.cn

**Keywords:** *κ*-*μ* shadowed fading, multi-parameter estimation, wireless sensor networks

## Abstract

In wireless sensor networks (WSNs), reliable link-quality estimation depends strongly on channel characteristics, which can be flexibly captured by the κ-μ shadowed fading model in complex closed metallic cavity (CMC) environments. However, a fundamental tension arises: conventional numerical methods for estimating the parameters of this model generally rely on the iterative solution of coupled nonlinear moment equations, which may introduce considerable computational cost and sensitivity to initialization and convergence settings, thereby limiting their applicability to rapid channel characterization in WSNs. We propose a closed-form multi-parameter estimation algorithm based on the method of moments that computes parameters directly without recourse to iterative numerical solvers. The key insight is that restructuring the normalized moment equations reveals a hidden quadratic structure, enabling explicit parameter extraction without iterative solver convergence requirements. Simulation results demonstrate sub-millisecond closed-form evaluation in the representative MATLAB implementation and substantial computational savings over the considered iterative solvers. Under the same prescribed composite parameter, it achieves essentially the same distribution-fitting accuracy as the corresponding constrained iterative estimator while requiring substantially less computation time. Furthermore, the reconstructed fading distributions provide accurate ergodic-capacity predictions, indicating the potential of the proposed method for computationally efficient link-quality characterization in WSNs.

## 1. Introduction

**Figure 1:**
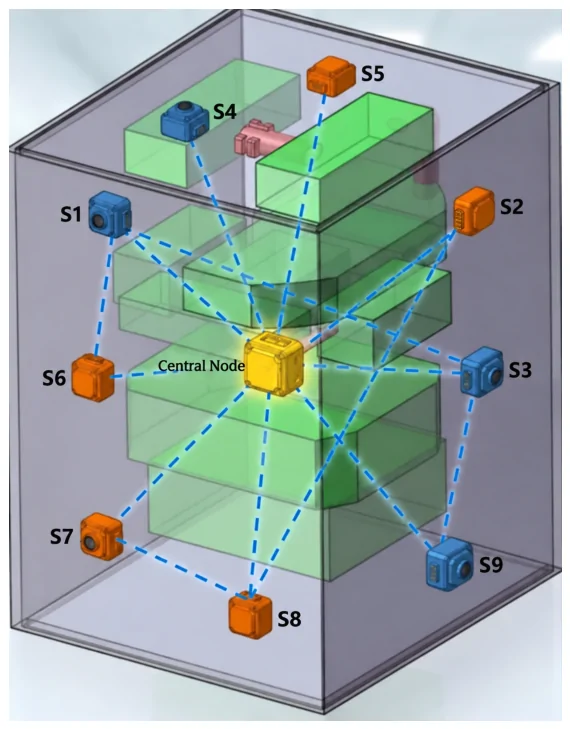
Representative CMC propagation scenario for a transportation-related WSN, illustrating multipath reflections and obstacle-induced shadowing within a confined metallic environment. S1–S9 denote representative sensor nodes.

Wireless sensor networks (WSNs) enable real-time monitoring and data acquisition in transportation systems, providing a scalable and flexible alternative to conventional wired sensing systems limited by cabling complexity, weight, and maintenance requirements [1,2]. In transportation-related closed metallic cavity (CMC) environments, such as automobile engine compartments and ultrahigh-speed train cabins, confined metallic boundaries and densely distributed internal structures create complex propagation conditions, as schematically illustrated in Figure 1. Multiple reflections from metallic surfaces and obstruction by internal components can produce clustered multipath and shadowing effects. Although deterministic ray-tracing methods are effective for site-specific propagation analysis when the geometry and material properties are accurately known, practical CMC channels may still exhibit statistical variations due to changes in the local propagation environment, obstruction conditions, and measurement uncertainty. Statistical channel models therefore remain useful for capturing such distribution-level variations and enabling computationally efficient link-quality characterization.

In such environments, conventional fading models such as Rayleigh and Rician may be insufficient to represent clustered multipath and shadowing effects, potentially leading to mismatches in statistical link-quality characterization. Accurate reconstruction of the instantaneous signal-to-noise ratio (SNR) distribution is therefore useful for evaluating communication-performance metrics and for supporting subsequent link-quality assessment. In this context, distribution-fitting fidelity can be as important as precise recovery of the individual fading parameters, particularly when different parameter combinations produce similar SNR distributions.

To characterize the complex fading phenomena in CMC environments, the κ-μ shadowed fading model [3] provides a unifying framework that captures channel characteristics such as multipath clustering while encompassing Rayleigh, Nakagami-*m*, and several other distributions as special or limiting cases. In recent years, the performance of wireless communication systems over κ-μ shadowed fading channels has been extensively investigated in terms of outage probability, error performance, ergodic capacity, and other system-level metrics [4,5,6,7]. In contrast to the extensive studies on statistical characterization and performance analysis, comparatively fewer works have addressed parameter estimation for the κ-μ shadowed fading model. Existing measurement-oriented studies have also investigated the applicability and statistical characteristics of the κ-μ shadowed model in different propagation environments. For example, Cañete et al. considered measured underwater acoustic channels, Cotton investigated second-order statistical characteristics of κ-μ shadowed fading, and Vega Sánchez et al. examined fading-model fitting for mmWave channels [8,9,10]. For the related κ-μ fading model, Tian et al. developed a multi-parameter estimation method and investigated a closed-form solution, while Xiang et al. further considered joint multiparameter estimation for wireless sensor network applications [11,12].

Nevertheless, existing measurement-oriented fitting studies often rely on numerical fitting procedures, whereas analytically tractable low-complexity approaches for simultaneous parameter recovery remain comparatively limited. This limitation motivates the development of a direct estimation framework that reduces the dependence on iterative numerical optimization. Accordingly, representative numerical root-finding, least-squares, local optimization, and stochastic optimization methods are considered as baselines in the numerical comparisons presented in Section 4. Moreover, the coupling among κ, μ, and *m* can lead to identifiability difficulties in recovering their individual physical values from measured data. Different parameter combinations may produce similar distributional characteristics, resulting in relatively flat or ill-conditioned regions of the estimation objective. Consequently, iterative algorithms may require additional iterations or converge to different feasible solutions depending on the initialization. These issues are particularly relevant to in-vehicle wireless sensor networks (IV-WSNs) and other time-varying CMC scenarios, where computationally efficient channel characterization is desirable for timely link-quality evaluation. Therefore, reducing the dependence on iterative numerical optimization is of practical interest for computationally efficient WSN channel characterization.

To address this, we propose a moment-based closed-form estimation framework that recasts the moment equations through normalized moments and variable substitutions into a solvable quadratic form, yielding an explicit solution without iterative refinement. Rather than pursuing exact physical parameter identification, our approach targets high-fidelity probability density function (PDF) reconstruction. This choice is motivated by a practical trade-off: conventional iterative estimators attempt to recover the individual parameters but may require repeated nonlinear evaluations and can exhibit sensitivity to initialization in ill-conditioned regions, which may limit their suitability for rapid or resource-constrained channel characterization. In contrast, accurate reconstruction of the fading distribution is directly relevant to link-quality evaluation and communication-performance prediction, since metrics such as ergodic capacity and outage probability are determined by the underlying SNR distribution rather than by the individual parameter values alone. By prioritizing distribution-fitting fidelity over parameter accuracy, the proposed method enables rapid, stable reconstruction with a substantially reduced computational cost.

The main contributions are as follows:

- We expose the hidden algebraic structure within the normalized moments of the κ-μ shadowed fading model and develop a reparameterization framework. This transforms the originally intractable coupled system into a form amenable to closed-form solution.
- We analyze the identifiability issue of the original three-parameter moment system and show that prescribing a composite parameter reduces the underdetermined problem to an explicit quadratic equation with a finite set of algebraic candidate solutions. Physical admissibility conditions are then used to screen these candidates, with an additional calibrated parameter-domain criterion applied when two physically admissible roots remain. This replaces iterative numerical solving in the core estimation stage with direct closed-form evaluation, enabling millisecond-level execution with substantially reduced computational cost.
- We evaluate the proposed method in terms of PDF fitting accuracy, Kullback–Leibler (KL) divergence, runtime, and capacity-prediction error, and compare it with representative numerical solvers. In addition, a same-*z* constrained Levenberg–Marquardt (LM) baseline is introduced to provide a direct comparison under the same prescribed composite parameter *z* and the same normalized-moment inputs. The results show that, for the representative out-of-domain comparison in which calibrated-domain screening is not applied, the proposed estimator recovers numerically equivalent solutions to the same-*z* constrained iterative method over the full Monte Carlo ensemble while substantially reducing the computational cost.

## 2. System Model and Problem Formulation

This section clearly defines the parameter estimation problem for the κ-μ shadowed fading channel model. The κ-μ shadowed fading model describes signal propagation in non-homogeneous environments, where the dominant component within each cluster is subject to random shadowing due to obstacles. According to this model, the PDF of the instantaneous signal-to-noise ratio (SNR) of the received signal can be expressed as [3]:

(1) $$f_{\rho }\left(\rho \right)=\frac{\theta_{1}^{m-\mu }}{\Gamma \left(\mu \right)\theta_{2}^{m}}\rho^{\mu -1}\times {e^{-\frac{\rho }{\theta_{1}}}}_{1}F_{1}\left(m,\mu ;\frac{\theta_{2}-\theta_{1}}{\theta_{1}\theta_{2}}\rho \right),$$

where $\theta_{1}=\bar{\rho }/\left(\mu +\mu \kappa \right)$ and $\theta_{2}=\left(\mu \kappa +m\right)\theta_{1}/m$. ρ is the instantaneous power SNR, $\rho =\bar{\rho }W/\bar{W}$, $\bar{\rho }=E\left(\rho \right)$, and $\bar{W}=E\left(W\right)=E\left(r^{2}\right)$. Here, κ is the ratio of the total dominant components to the total scattered wave power, μ>0 denotes the number of multipath clusters (which may take non-integer values [13]), and *m* characterizes the shadowing severity (larger *m* implies lighter shadowing).

Based on the PDF of the κ-μ shadowed fading model, its moment-generating function (MGF) can be obtained as in [14]:

(2) $$MGF_{\rho }\left(s\right)=\int_{0}^{+\infty }e^{s\rho }f_{\rho }\left(\rho \right)\;d\rho ={\left(-1\right)}^{\mu }\frac{{\left(s\theta_{1}-1\right)}^{m-\mu }}{{\left(s\theta_{2}-1\right)}^{m}}.$$

This model encompasses several classical fading distributions, including the κ-μ, Rician shadowed, Rician, Nakagami-*m*, Rayleigh, and one-sided Gaussian distributions, as special or limiting cases [3].

The parameter estimation for the κ-μ shadowed fading model employs a method based on moment functions. By leveraging the properties of the MGF, the *j*-th order moment corresponding to the instantaneous power SNR ρ in the κ-μ shadowed fading model can be derived, i.e., the *j*-th derivative of the MGF at *s* = 0. For (2), setting *s* = 0 and taking the *j*-th derivative yields the expression for the moments of the κ-μ shadowed fading model:

(3) $$\begin{array}{c} E\left(\rho^{j}\right)={\left(-1\right)}^{j}{\left(\frac{\bar{\rho }}{\mu (1+\kappa )}\right)}^{j}\frac{\Gamma (m-\mu +1)}{\Gamma (m-\mu +1-j)}\cdot_{2}F_{1}\left(-j,m;m-\mu +1-j;\frac{\mu \kappa +m}{m}\right),\\ \;j\in Z^{+} \end{array}$$

where *j* is a positive integer and F12(·,·;·;·) denotes the Gauss hypergeometric function [15]. In this work, only the low-order moments with j=1,2,3 are required. Since the first parameter of F12 is −j, the corresponding hypergeometric series terminates after j+1 terms and can therefore be expressed as a finite polynomial. The Gauss hypergeometric function is defined as:

(4) F12(a,b;c;z)=∑n=0∞(a)n(b)n(c)nznn!.

Here, ${\left(a\right)}_{n}$ denotes the Pochhammer symbol (rising factorial), defined as

$${\left(a\right)}_{n}=\left\{\begin{array}{cc} 1, & n=0,\\ a(a+1)\cdots (a+n-1), & n\geq 1. \end{array}\right.$$

Let j=1,2,3 and expand the moments:

(5) $$E\left(\rho \right)=-\theta_{1}\left(m-\mu \right)+\theta_{2}m,$$

(6) $$\begin{array}{c} E\left(\rho^{2}\right)=\theta_{1}^{2}\left(m-\mu \right)\left(m-\mu -1\right)-2\theta_{1}\theta_{2}m\left(m-\mu \right)+\theta_{2}^{2}m\left(m+1\right), \end{array}$$

(7) $$\begin{array}{cc} E(\rho^{3})= & -\theta_{1}^{3}\left(m-\mu \right)\left(m-\mu -1\right)\left(m-\mu -2\right)+3\theta_{1}^{2}\theta_{2}m\left(m-\mu \right)\left(m-\mu -1\right)\\ \; & -3\theta_{1}\theta_{2}^{2}m\left(m+1\right)\left(m-\mu \right)+\theta_{2}^{3}m\left(m+1\right)\left(m+2\right), \end{array}$$

where $\theta_{1}=\bar{\rho }/\left(\mu +\mu \kappa \right)$ and $\theta_{2}=\left(\mu \kappa +m\right)\theta_{1}/m$. Equations (5)–(7) give the first three raw moments of the κ-μ shadowed distribution. However, after normalization by the average SNR, the first-moment relation reduces to the identity $E\left[\rho \right]=\bar{\rho }$ and therefore does not provide an independent shape constraint on κ, μ, and *m*. Consequently, the normalized second- and third-order moments provide only two independent shape equations for the three coupled channel parameters. Additional prior information is therefore required to reduce the underdetermined parameter-recovery problem. Section 3 addresses this issue by introducing the composite parameter z=κμ/m as pre-calibrated side information, which reduces the normalized-moment system to a quadratic equation with a finite set of candidate solutions. Physical admissibility conditions and, when necessary, a calibrated parameter-domain criterion are then used to determine the final estimate.

## 3. Parameter Estimation Algorithm

The proposed estimation framework is built upon a central observation: while the original parameter space (κ,μ,m) suffers from severe nonlinear coupling, making direct inversion of the moment equations intractable, the normalized moments $\left(Q_{2},Q_{3}\right)$ exhibit a hidden quadratic structure when expressed in suitably chosen composite variables.

To exploit this structure, we introduce the following reparameterization. Let P=μ(1+κ),z=κμ/m,d=m−μ, where these composite variables serve to absorb the nonlinear coupling among κ, μ, and *m*, transforming the original three-parameter system into a more tractable form. In the proposed framework, the aggregate parameter *z* is treated as an environment-dependent composite quantity that can be obtained from prior calibration and held fixed over a subsequent estimation interval. In practice, the prescribed value of *z* can be obtained during an offline calibration stage using a sufficiently long reference measurement collected under representative environmental conditions. The full κ-μ shadowed model can first be fitted using an offline numerical estimator, after which z=κμ/m is computed and stored as an environment-specific calibration quantity. During subsequent online estimation intervals, the proposed closed-form estimator uses this calibrated value without repeating the computationally expensive three-parameter fitting procedure. Prescribing *z* supplies additional prior information and reduces the underdetermined normalized-moment system to a quadratic equation in *P* with a finite set of algebraic candidate solutions. The resulting candidate solutions are subsequently screened using the admissibility and ambiguity-resolution procedure described below to determine the final estimate whenever a unique solution can be identified. The robustness of the estimator to mismatch in the prescribed value of *z* is investigated separately in Section 4.4.

The estimation then proceeds in three steps:

1. Normalization: Compute the sample moments ${\hat{\mu }}_{1}$, ${\hat{\mu }}_{2}$, ${\hat{\mu }}_{3}$ from received samples, and form the normalized moments $Q_{2}={\hat{\mu }}_{2}/{\hat{\mu }}_{1}^{2}$ and $Q_{3}={\hat{\mu }}_{3}/{\hat{\mu }}_{1}^{3}$. This eliminates dependence on the average $\bar{\rho }$, reducing the problem dimensionality.
2. Reparameterization and variable substitution: Substitute the original parameters with *P*, *z*, and *d* in the normalized moment expressions. The resulting equations decouple the nonlinear interactions among the original variables.
3. Closed-form solution: With *z* fixed, the system reduces to a quadratic equation in *P*, whose explicit solution is given by (18). The remaining parameters *d*, μ, *m*, and κ are then recovered sequentially via (24)–(27).

Before implementation, we introduce the actual estimates of the moments obtained from sample moments:

(8) $${\hat{\mu }}_{1}=\frac{1}{N}\sum_{i=1}^{N}|\rho_{i}|,\;{\hat{\mu }}_{2}=\frac{1}{N}\sum_{i=1}^{N}{\left|\rho_{i}\right|}^{2},\;{\hat{\mu }}_{3}=\frac{1}{N}\sum_{i=1}^{N}{\left|\rho_{i}\right|}^{3},$$

where $\rho_{i}$ (i=1,…,N) represent samples generated according to the theoretical PDF using the Monte Carlo acceptance-rejection method in [16]. According to the strong law of large numbers [17], the sample moments converge almost surely to their corresponding theoretical moments as the sample size increases. Consequently, the proposed moment-based estimates are expected to approach the corresponding population-moment solution as *N* increases, provided that a physically admissible solution exists. This finite-sample convergence behavior is further investigated numerically through the sample-size sensitivity analysis in Section 4.5.

First, derive the normalized moments $Q_{2}$ and $Q_{3}$ using (3), (5)–(7), where $E\left[\rho \right]=\bar{\rho }$, $Q_{2}=E\left[\rho^{2}\right]/{\left(E\left[\rho \right]\right)}^{2}$, $Q_{3}=E\left[\rho^{3}\right]/{\left(E\left[\rho \right]\right)}^{3}$, as follows:

(9) Q2=1[μ(1+κ)]2[(m−μ)(m−μ−1)F12−2,m;m−μ−1;μκ+mm],Q3=−1[μ(1+κ)]3[(m−μ)(m−μ−1)(m−μ−2)F12−3,m;m−μ−2;μκ+mm].

Observing this system of equations, consider using variable substitution to re-express them into a simpler form.

Let P=μ(1+κ), z=κμ/m, d=m−μ. After using series representation of the Gauss hypergeometric function and simplifying, we obtain:

(10) $$\left\{\begin{array}{c} \begin{array}{cc} Q_{2}=\frac{1}{P^{2}}[ & dz+\left(z+1\right)\left(mz+m-d\right)+{\left(mz+m-d\right)}^{2}], \end{array}\\ \begin{array}{cc} Q_{3}=-\frac{1}{P^{3}}[ & -4dz-2dz^{2}+\left(mz+m-d\right)\left(-2-4z-2z^{2}-3dz\right)\\ \; & -{\left(mz+m-d\right)}^{2}\left(3+3z\right)-{\left(mz+m-d\right)}^{3}], \end{array} \end{array}\right.$$

where P=μ(1+κ)=mz+m−d, we can obtain simple expressions in terms of *P*, *z*, and *d*:

(11) $$\left\{\begin{array}{c} Q_{2}=1+\frac{z+1}{P}+\frac{zd}{P^{2}},\\ Q_{3}=1+\frac{3(z+1)}{P}+\frac{3zd+2{\left(z+1\right)}^{2}}{P^{2}}+\frac{2zd(z+2)}{P^{3}}. \end{array}\right.$$

Since three composite unknowns, *P*, *z*, and *d*, correspond to only two normalized moment equations, the resulting system is underdetermined. We therefore introduce one additional piece of prior information by treating z=κμ/m as a pre-calibrated composite parameter and explicitly solve for the remaining two unknowns, *P* and *d*.

The choice of *z* is motivated by the fact that it combines the effects of the dominant-to-scattered power ratio, the number of multipath clusters, and the shadowing parameter into a single composite quantity. Compared with treating κ, μ, and *m* as three independent unknowns, prescribing *z* supplies one additional piece of prior information and reduces the underdetermined normalized-moment system to a quadratic equation with a finite set of algebraic candidate solutions. However, prescribing *z* does not necessarily guarantee a unique physically admissible solution for every finite-sample realization. When both algebraic roots satisfy the physical admissibility conditions, an additional calibrated parameter-domain criterion is used to resolve the remaining ambiguity, as described below. Importantly, *z* is not estimated from the same sample realization used to evaluate the proposed estimator. The influence of mismatch between the prescribed and true values of *z* is independently evaluated in Section 4.4.

Thus, given $Q_{2}$, $Q_{3}$, and the prescribed value of *z*, explicit expressions for *P* and *d* can be derived from (11).

To eliminate the constant and 1/P terms, define

(12) $$D=Q_{3}-3Q_{2}+2.$$

Using (11), *D* can be expressed as

(13) $$D=\frac{2{\left(z+1\right)}^{2}}{P^{2}}+\frac{2zd(z+2)}{P^{3}}.$$

Meanwhile, the expression of $Q_{2}$ gives

(14) $$Q_{2}-1=\frac{z+1}{P}+\frac{zd}{P^{2}},$$

and hence

(15) $$zd=\left(Q_{2}-1\right)P^{2}-\left(z+1\right)P.$$

Substituting (15) into (13) yields

(16) $$DP^{2}-2\left(z+2\right)\left(Q_{2}-1\right)P+2\left(z+1\right)=0.$$

For convenience, define

(17) $$\Delta ={\left(z+2\right)}^{2}{\left(Q_{2}-1\right)}^{2}-2\left(z+1\right)D,$$

where Δ is one quarter of the conventional discriminant of the quadratic equation. For $|D|>\varepsilon_{D}$ and Δ≥0, the two algebraic candidate roots are

(18) $$P_{\pm }=\frac{\left(z+2\right)\left(Q_{2}-1\right)\pm \sqrt{\Delta }}{D}.$$

It should be emphasized that the two algebraic roots in (18) are only candidate solutions. A candidate root is physically meaningful only if the recovered κ-μ shadowed parameters satisfy

(19) P>0,μ>0,m>0,κ≥0.

For z>0, solving the $Q_{2}$ equation for *d* gives

(20) $$d=\frac{\left(Q_{2}-1\right)P^{2}-\left(z+1\right)P}{z}.$$

Substitution of (20) into

$$\mu =\frac{P-dz}{z+1}$$

gives

(21) $$\mu =\frac{P\left[\left(z+2\right)-\left(Q_{2}-1\right)P\right]}{z+1}.$$

Similarly, using m=d+μ yields

(22) $$m=\frac{P\left[(Q_{2}-1)P-1\right]}{z(z+1)}.$$

Since z>0 and P>0, the conditions μ>0 and m>0 require

$$(Q_{2}-1)P<z+2$$

and

$$(Q_{2}-1)P>1,$$

respectively. Therefore, for $Q_{2}>1$, the physically admissible interval of *P* is

(23) $$\frac{1}{Q_{2}-1}<P<\frac{z+2}{Q_{2}-1}.$$

Moreover, since

$$\kappa =\frac{zm}{\mu },$$

the conditions z>0, μ>0, and m>0 automatically imply κ>0.

Accordingly, both algebraic roots $P_{+}$ and $P_{-}$ in (18) are treated as candidate solutions. For each candidate, the corresponding *d*, μ, *m*, and κ are recovered and checked against the basic physical admissibility conditions in (19) and (23). If exactly one candidate is admissible, it is selected; if neither is admissible, the realization is classified as infeasible. If both candidates are physically admissible, the calibrated parameter domain used in the numerical experiments, 0.1≤κ≤2, 1.5≤μ≤5, and 2≤m≤6, is used to resolve the ambiguity. A candidate outside this domain is discarded; if both candidates remain within the domain, the realization is classified as ambiguous. For parameter settings outside the calibrated domain, this domain-based criterion is not applied. In such cases, the candidates are screened only according to the physical admissibility conditions: if exactly one physically admissible candidate remains, it is accepted; if no physically admissible candidate exists, the realization is classified as infeasible; and if two physically admissible candidates remain, the realization is classified as unresolved. It should therefore be emphasized that the calibrated parameter domain serves as an additional ambiguity-resolution criterion beyond the prescribed value of *z* only for parameter settings to which the calibration range is applicable. The role of the prescribed *z* is to reduce the original underdetermined moment system to a finite set of closed-form candidates, whereas uniqueness is determined primarily by the physical admissibility conditions and, for in-domain cases when necessary, by calibrated parameter-domain screening. For out-of-domain cases, no incompatible domain constraint is imposed, and unresolved multiple-root cases are explicitly retained as such.

For the degenerate case D=0, (16) reduces to

$$-2\left(z+2\right)\left(Q_{2}-1\right)P+2\left(z+1\right)=0,$$

which gives

$$P=\frac{z+1}{\left(z+2\right)\left(Q_{2}-1\right)}.$$

For z>0 and $Q_{2}>1$, however,

$$\frac{z+1}{\left(z+2\right)\left(Q_{2}-1\right)}<\frac{1}{Q_{2}-1},$$

and hence this solution violates the admissibility condition in (23). Therefore, D=0 does not yield a physically admissible solution for the z>0 branch. In numerical implementation, a small threshold $\varepsilon_{D}$ is used to identify near-degenerate cases and avoid unstable division by a near-zero value of *D*.

In finite-sample implementations, sampling fluctuations and floating-point round-off may also lead to a negative discriminant. A numerical tolerance $\varepsilon_{\Delta }$ is therefore introduced. If

$$-\varepsilon_{\Delta }\leq \Delta <0,$$

the small negative value is treated as numerical round-off and Δ is set to zero, resulting in a repeated real root. If

$$\Delta <-\varepsilon_{\Delta },$$

no real-valued solution exists for the observed normalized moments under the prescribed value of *z*, and the current realization is declared infeasible rather than artificially forcing a real-valued solution.

For the selected admissible root $\hat{P}$, the auxiliary parameter *d* is estimated as

(24) $$\hat{d}=\frac{\left(Q_{2}-1\right){\hat{P}}^{2}-\left(z+1\right)\hat{P}}{z},\;z>0.$$

The original parameters are subsequently recovered as

(25) $$\hat{\mu }=\frac{\hat{P}-z\hat{d}}{z+1},$$

(26) $$\hat{m}=\hat{d}+\hat{\mu },$$

and

(27) $$\hat{\kappa }=\frac{z\hat{m}}{\hat{\mu }}.$$

Finally, the limiting case z=0 is treated separately because (24) is singular at z=0. Since

$$z=\frac{\kappa \mu }{m},$$

with μ>0 and m>0, z=0 corresponds to κ=0. In this case, (11) reduces to

(28) $$Q_{2}=1+\frac{1}{P},\;Q_{3}=1+\frac{3}{P}+\frac{2}{P^{2}}.$$

Since P=μ when κ=0, we obtain

(29) $$\hat{\kappa }=0,\;\hat{\mu }=\frac{1}{Q_{2}-1}.$$

The corresponding normalized moments satisfy

(30) $$Q_{3}=2Q_{2}^{2}-Q_{2}.$$

When κ=0, the dominant component vanishes and the shadowing parameter *m* no longer affects the resulting limiting distribution. Therefore, *m* is structurally unidentifiable from the considered moments in this boundary case and is not artificially assigned through (24).

## 4. Simulation Verification

### 4.1. Simulation Setup and Reproducibility

All simulations were implemented in MATLAB R2024b and executed on a computer equipped with an Intel(R) Core(TM) Ultra 7 255HX processor operating at 2.40 GHz and 16 GB of RAM. Unless otherwise specified, the average SNR was normalized to $\bar{\rho }=1$. To evaluate the estimation performance under representative channel conditions, the parameters of the κ-μ shadowed model were selected from

(31) κ∈[0.1,2],μ∈[1.5,5],m∈[2,6].

For each parameter configuration, 100 independent Monte Carlo trials were performed, with N=5000 samples generated in each trial. A fixed random seed was used for the generation of parameter configurations to ensure reproducibility. Within each Monte Carlo trial, all estimation methods were evaluated using exactly the same channel-sample realization, thereby avoiding performance differences caused by different data realizations.

The κ-μ shadowed samples were generated using an acceptance–rejection sampling procedure according to the target PDF. For each parameter configuration, the average SNR was normalized to $\bar{\rho }=1$, and the sampling interval was set to $0<p\leq p_{\max}$, with $p_{\max}=6$. To construct the rejection envelope, the theoretical PDF was first evaluated at 1000 uniformly spaced points over $\left[0.01,p_{\max}\right]$. The envelope height was then chosen as

$$M=1.05\max_{p\in G}f_{\rho }\left(p;\kappa ,\mu ,m\right),$$

where G denotes the numerical grid. The factor 1.05 provides a small safety margin above the numerically determined maximum. Candidate points were independently generated according to

$$p_{c}∼U\left(0,p_{\max}\right),\;y_{c}∼U\left(0,M\right),$$

and $p_{c}$ was accepted when

$$y_{c}\leq f_{\rho }\left(p_{c};\kappa ,\mu ,m\right).$$

Candidate generation was repeated until *N* accepted samples were obtained. A numerical verification over the considered parameter range showed that the probability mass beyond $p_{\max}=6$ remained below $4.31\times 10^{-4}$, indicating that the finite sampling interval retains more than 99.95% of the probability mass.

For each sample realization, the first four raw sample moments were calculated as

(32) $$U_{j}=\frac{1}{N}\sum_{i=1}^{N}p_{i}^{j},\;j=1,2,3,4.$$

All compared methods used moments calculated from the same sample realization. For the numerical baseline methods, the corresponding moment equations were expressed through the residual vector

(33) $$F\left(x\right)=\left[\begin{array}{c} F_{2}\left(x\right)\\ F_{3}\left(x\right)\\ F_{4}\left(x\right) \end{array}\right],\;x={\left[\begin{array}{ccc} m & \mu & \kappa \end{array}\right]}^{T},$$

where $F_{j}\left(x\right)$ denotes the residual between the theoretical and sample moment of the corresponding order. The optimization-based methods minimize

(34) $$J\left(x\right)={∥F\left(x\right)∥}_{2}^{2},$$

whereas the root-finding methods directly solve the corresponding nonlinear moment equations. Thus, all methods are evaluated using the same sample realization. The conventional numerical baselines solve the original three-parameter moment system, whereas the proposed estimator uses two normalized moment equations together with the pre-calibrated composite parameter *z*. To isolate the effect of this prescribed side information, a same-*z* constrained LM baseline is additionally considered using the same *z*, $Q_{2}$, and $Q_{3}$ as the proposed estimator. The comparison therefore evaluates the computational and distribution-fitting trade-off between direct constrained estimation and conventional numerical solution of the original parameter system.

The nonlinear least-squares method was implemented using lsqnonlin, the trust-region and Levenberg–Marquardt methods using fsolve, and the quasi-Newton method using fminunc. For the bounded numerical optimization methods, the parameter search interval was set to

(35) 0.01≤m,μ,κ≤100.

Unless otherwise specified, the stopping tolerances, maximum numbers of function evaluations, and convergence criteria of the deterministic numerical solvers were kept at their default MATLAB R2024b settings. For the stochastic solvers, genetic algorithm (GA) employed a population size of 100 and a maximum of 50 generations, whereas simulated annealing (SA) used a maximum of 500 iterations. Each stochastic optimizer was executed once for each independent Monte Carlo data realization.

For the deterministic iterative methods, including nonlinear least-squares, trust-region, Levenberg–Marquardt, and fminunc, the initial point was obtained from the solution returned by MATLAB’s solve function for the same set of sample moments, i.e., $x_{0}={\left[m_{\mathrm{solve}},\mu_{\mathrm{solve}},\kappa_{\mathrm{solve}}\right]}^{T}$. The same solve-based initialization was used for these four methods, and no multi-start procedure was employed. GA used its population-based initialization, whereas SA was initialized randomly within the prescribed parameter bounds using a fixed random-number-generator state.

The proposed estimator first constructs $Q_{2}$ and $Q_{3}$ from the observed samples and then uses a prescribed value of the composite quantity *z*, representing prior calibration information, together with the derived closed-form expressions to recover $\hat{\kappa }$, $\hat{\mu }$, and $\hat{m}$. Unless otherwise specified, the nominal calibration case $z_{\mathrm{used}}=z_{\mathrm{true}}$ is considered to isolate the intrinsic estimation behavior of the closed-form solution. The robustness to imperfect calibration, i.e., $z_{\mathrm{used}}\neq z_{\mathrm{true}}$, is separately evaluated in Section 4.4. To provide a direct comparison under the same prescribed composite parameter *z* and the same normalized sample moments $Q_{2}$ and $Q_{3}$, an additional same-*z* constrained LM baseline is introduced. With *z* fixed, the relation z=κμ/m is imposed during numerical optimization. Therefore, the same-*z* constrained LM and the proposed estimator share the same prescribed-*z* constraint and the same normalized-moment inputs, while differing in their solution strategies: the former relies on iterative numerical optimization, whereas the latter directly evaluates the derived closed-form candidates followed by the prescribed admissibility procedure. Unlike the proposed root-selection procedure, the same-*z* constrained LM does not employ the calibrated parameter-domain criterion. Specifically, the same-*z* constrained LM minimizes the squared residuals of the two normalized-moment equations,

$$J_{\mathrm{cLM}}\left(y\right)={\left[Q_{2}\left(y;z\right)-{\hat{Q}}_{2}\right]}^{2}+{\left[Q_{3}\left(y;z\right)-{\hat{Q}}_{3}\right]}^{2},$$

where $y={\left[\log\mu ,\log m\right]}^{T}$, $\mu =\exp(y_{1})$, $m=\exp(y_{2})$, and the prescribed-*z* constraint is imposed through κ=zm/μ. The logarithmic parameterization guarantees μ>0 and m>0, so no additional box bounds are imposed. A fixed, physically valid initialization of $\left(\mu_{0},m_{0}\right)=\left(2,5\right)$ is used independently of the proposed closed-form estimate, and no multi-start procedure is employed. MATLAB lsqnonlin with the LM algorithm is used with MaxIterations = 500, MaxFunctionEvaluations = 3000, FunctionTolerance = $10^{-12}$, and StepTolerance = $10^{-12}$. For numerical feasibility assessment, solver-reported success and physical admissibility are recorded separately. Solver-reported success is determined according to the termination flag returned by the corresponding MATLAB solver, whereas a returned estimate is regarded as physically admissible if its parameters are finite and satisfy κ≥0, μ>0, and m>0. A non-success solver flag does not imply the absence of a returned parameter vector; physical admissibility is therefore assessed separately. Therefore, the proposed estimator does not require iterative numerical optimization, parameter initialization, a stopping tolerance, or a maximum number of iterations.

For the computational-time comparison, channel-sample generation was excluded because it is common to all estimation methods. The common sample-moment calculations were also performed before the solver-specific timing operation. For the same-*z* constrained LM baseline, the reported runtime includes only the constrained iterative optimization stage after $Q_{2}$, $Q_{3}$, and the prescribed *z* have been provided, thereby ensuring a direct timing comparison with the proposed closed-form estimator using the same normalized-moment inputs and prescribed composite parameter. For nonlinear least-squares, trust-region, Levenberg–Marquardt, and fminunc, the reported runtimes include only their respective optimization stages and exclude the preceding solve step used to obtain the common initialization. Therefore, their reported runtimes should be interpreted as solver-stage computational costs under a precomputed initialization rather than as end-to-end runtimes. This timing protocol provides the same sample-derived inputs and preprocessing conditions for the corresponding solver-stage comparisons while avoiding the inclusion of common computational operations in the reported runtimes.

For each numerical baseline, solver-reported success status and physical admissibility of the returned estimate are recorded separately. Solver-reported success is determined from the solver-specific termination flag, whereas physical admissibility requires finite recovered parameters satisfying κ≥0, μ>0, and m>0. Non-finite or physically inadmissible outputs are recorded as NaN and excluded from conditional error averaging. Because solver-specific termination criteria differ among deterministic and stochastic optimizers, the solver-reported success rate and physical-output rate are reported separately rather than being combined into a single threshold-dependent validity measure. Numerical residual statistics are additionally examined to characterize how closely the returned estimates satisfy the corresponding moment equations.

### 4.2. Computational Efficiency Analysis

For the computational-efficiency comparison, the sample size is set to N=5000. The runtime per estimation for the proposed method and the numerical baseline methods described in Section 4.1 is summarized in Table 1.

As shown in Table 1, the proposed closed-form estimator requires only 0.180 ms per estimation, substantially less than the considered numerical methods. More importantly, the newly introduced same-*z* constrained LM baseline provides a direct computational comparison under the same prescribed *z* and the same normalized-moment inputs. The same-*z* constrained LM requires 9.729 ms, whereas the proposed estimator requires only 0.180 ms, corresponding to an approximately 54-fold reduction in computational time. This result demonstrates the computational benefit of replacing iterative numerical optimization with direct closed-form evaluation.

### 4.3. Estimation Accuracy Analysis

The PDF curve of the κ-μ shadowed fading model is determined by the parameters κ, μ, and *m*. However, these three parameters do not have a straightforward linear relationship, so the accuracy of the estimation algorithm cannot be simply analyzed by the deviation between estimated and theoretical values. Therefore, distribution-reconstruction accuracy is evaluated using two complementary metrics: the PDF root mean square error (RMSE) and the KL divergence. The PDF RMSE quantifies pointwise fitting error over a common SNR grid, whereas the KL divergence measures the discrepancy between the normalized true and reconstructed distributions.

The RMSE calculation formula is:

(36) $$RMSE=\sqrt{\sum_{i=1}^{n}{\left(f_{\mathrm{PDF}}\left(\kappa ,\mu ,m,\rho_{i}\right)-f_{\mathrm{PDF}}\left(\hat{\kappa },\hat{\mu },\hat{m},\rho_{i}\right)\right)}^{2}/n}.$$

The KL divergence is evaluated as $D_{\mathrm{KL}}\left(f_{\mathrm{true}}∥f_{\mathrm{est}}\right)$ over a common SNR grid. Specifically, both the true and reconstructed PDFs are evaluated over the complete numerical support $\left[10^{-8},15\bar{\rho }\right]$ using 10,000 uniformly spaced grid points. To avoid numerical singularities caused by floating-point underflow, non-positive or numerically underflowed density values are replaced by a floor of $10^{-12}$. Both PDFs are then renormalized over the complete common grid after application of the numerical floor, and the KL divergence is evaluated by numerical integration without discarding individual grid points.

For the representative channel configuration with N=5000, κ=1, μ=2, and m=10, the PDF fitting RMSEs of the different estimation methods are reported in Table 1. It should be noted that m=10 lies outside the calibrated parameter domain 2≤m≤6 used for ambiguity resolution in the in-domain experiments. Therefore, the calibrated-domain criterion is not applied in this representative out-of-domain comparison. Instead, the algebraic candidates of the proposed estimator are screened only according to the physical admissibility conditions. If exactly one physically admissible candidate remains, it is accepted; if no physically admissible candidate exists, the realization is classified as infeasible; and if two physically admissible candidates remain, the realization is classified as unresolved rather than forcing a selection using an incompatible calibration range. In this case, the same-*z* constrained LM and the proposed closed-form estimator use the same prescribed *z* and the same normalized moments $Q_{2}$ and $Q_{3}$. Both methods achieve the same PDF RMSE of 0.005087 to the reported precision. The same-*z* constrained LM converges to $\left(\hat{\kappa },\hat{\mu },\hat{m}\right)=\left(1.3237,1.8153,12.0142\right)$, which agrees with the proposed estimator to the reported precision. This agreement verifies the consistency between the closed-form and iterative solutions under the same prescribed-*z* constraint for this representative realization. The PDF RMSE values in Table 1 are computed for the reported representative realization, whereas the solver-termination and physical-admissibility statistics over the full 100-realization Monte Carlo ensemble are reported separately in Table 2.

To further assess numerical feasibility beyond this single representative realization, an additional 100-realization Monte Carlo comparison was conducted for the same parameter configuration (κ,μ,m)=(1,2,10) with N=5000. Solver-reported success and physical admissibility were recorded separately for each numerical method. The solver-reported success rates of solve, nonlinear least squares, trust-region, LM, fminunc, GA, SA, and the same-*z* constrained LM were 8%, 100%, 90%, 2%, 66%, 0%, 0%, and 100%, respectively. The proposed estimator does not involve an iterative solver or a solver-reported success flag and produced a physically admissible estimate in all 100 realizations. For this out-of-domain configuration, no realization produced two physically admissible algebraic roots; hence, the proposed estimator obtained a unique physical solution in all 100 trials without invoking calibrated-domain screening. Moreover, the proposed estimator and the same-*z* constrained LM both produced physically admissible estimates in all 100 realizations. Defining numerical agreement by a relative parameter-vector difference below $10^{-6}$, the two methods agreed in 100/100 realizations (100%). The median and maximum relative parameter differences were $1.07\times 10^{-14}$ and $1.26\times 10^{-13}$, respectively, confirming numerical equivalence of the two solution strategies over the complete Monte Carlo ensemble for this out-of-domain test case.

**Table 1:** Comparison of PDF fitting RMSE and computational time for different estimation algorithms.

Algorithm Name	PDF RMSE	Response Time (ms)
Solve function	0.038363	4054.2
Least squares solution	0.008355	46.4
Trust-region algorithm	0.011814	21.0
Levenberg–Marquardt	0.010648	11.9
Fminunc	0.023767	21.0
Genetic algorithm	0.010996	65.5
Simulated annealing	NaN	217.9
Same-*z* constrained LM	0.005087	9.729
Proposed closed-form estimator	0.005087	0.180

**Table 2:** Numerical feasibility in the 100-realization Monte Carlo comparison for (κ,μ,m)=(1,2,10).

Algorithm Name	Solver-Reported Success Rate	Physically Admissible Output Rate
Solve function	8%	8%
Least squares solution	100%	100%
Trust-region algorithm	90%	100%
Levenberg–Marquardt	2%	100%
Fminunc	66%	100%
Genetic algorithm	0%	100%
Simulated annealing	0%	100%
Same-*z* constrained LM	100%	100%
Proposed closed-form estimator	N/A	100%

Note: Solver-reported success is determined from the MATLAB solver flag, whereas physical admissibility is assessed independently.

As an additional diagnostic, the moment-equation residuals of the physically admissible outputs were examined. For the conventional baselines, relative residuals of the second- to fourth-order raw-moment equations were used, whereas the same-*z* constrained LM and the proposed estimator were evaluated using the normalized $Q_{2}$-$Q_{3}$ residuals corresponding to their estimation equations. Because these methods solve different moment systems and MATLAB solvers employ method-specific termination criteria, no arbitrary universal residual threshold was imposed to redefine numerical validity. In particular, the median normalized $Q_{2}$-$Q_{3}$ residuals of the same-*z* constrained LM and the proposed estimator were on the order of $10^{-16}$, confirming that both methods satisfy their common prescribed-*z* moment equations to numerical precision.

To quantify the practical role of the calibrated parameter-domain criterion, the root-selection outcomes were additionally recorded over the 10 in-domain parameter configurations listed in Table 3, with 100 Monte Carlo realizations for each configuration. Among the resulting 1000 realizations, 754 (75.4%) yielded a unique solution using the physical admissibility conditions alone, whereas 212 (21.2%) produced two distinct physically admissible roots and therefore required the calibrated parameter-domain criterion. Among these 212 cases, the domain criterion uniquely resolved 179 cases (84.43%), while 33 cases (15.57%) remained ambiguous. The remaining 34 realizations (3.4%) did not yield real algebraic candidates because the discriminant was below the prescribed numerical tolerance. These results confirm that prescribing *z* reduces the underdetermined normalized-moment system to a finite set of algebraic candidates but does not by itself guarantee a unique estimate for every finite-sample realization.

**Figure 2:**
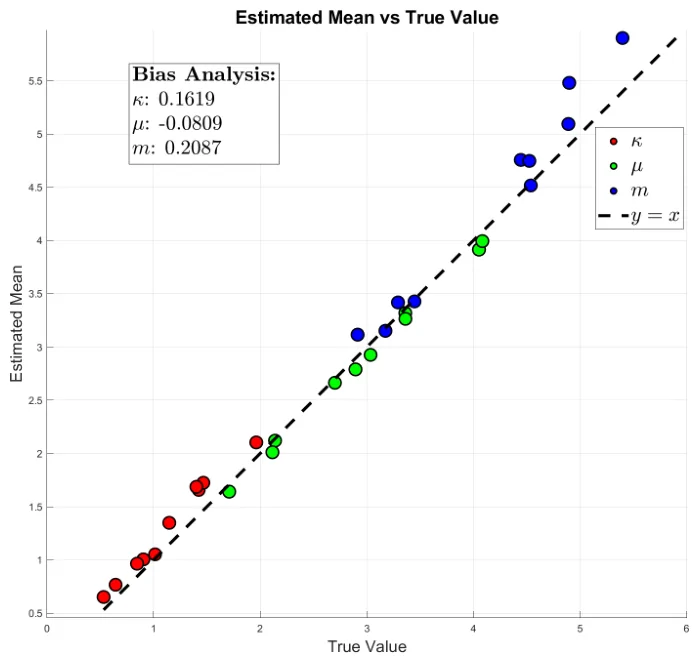
Estimated mean versus true values for κ, μ, and *m*. The dashed line represents y=x, and the inset reports the corresponding average estimation biases.

For the representative parameter range considered in the simulations, κ, μ, and *m* are randomly selected within κ∈[0.1,2], μ∈[1.5,5], and m∈[2,6], respectively. Each reported result is the average over 100 independent Monte Carlo trials, with N=5000 samples per trial. Figure 2 compares the mean estimated parameters with their corresponding true values. The dashed line represents the ideal relation y=x. The estimated values generally follow the variation trends of the true parameters, although some deviations remain for certain configurations, particularly for *m*. The average estimation biases are 0.1619, −0.0809, and 0.2087 for κ, μ, and *m*, respectively. These results indicate that the proposed estimator captures the overall variation in the underlying channel parameters over the considered range, while the remaining deviations reflect the nonlinear coupling and identifiability difficulty among κ, μ, and *m*.

Nevertheless, parameter accuracy alone does not fully determine the quality of the reconstructed fading distribution. As summarized in Table 3, the PDF RMSE remains between approximately 0.0040 and 0.0079 over the considered parameter sets. This indicates that moderate deviations in the individual recovered parameters can be partially compensated by changes in the remaining parameters, allowing different physically admissible parameter combinations to produce very similar fading distributions. Therefore, the parameter deviations observed in Figure 2 should be interpreted together with the distribution-fitting errors in Table 3. The relatively small PDF RMSE values indicate that the proposed estimator preserves the overall statistical characteristics of the target fading distribution despite the remaining parameter-estimation errors.

**Figure 3:**
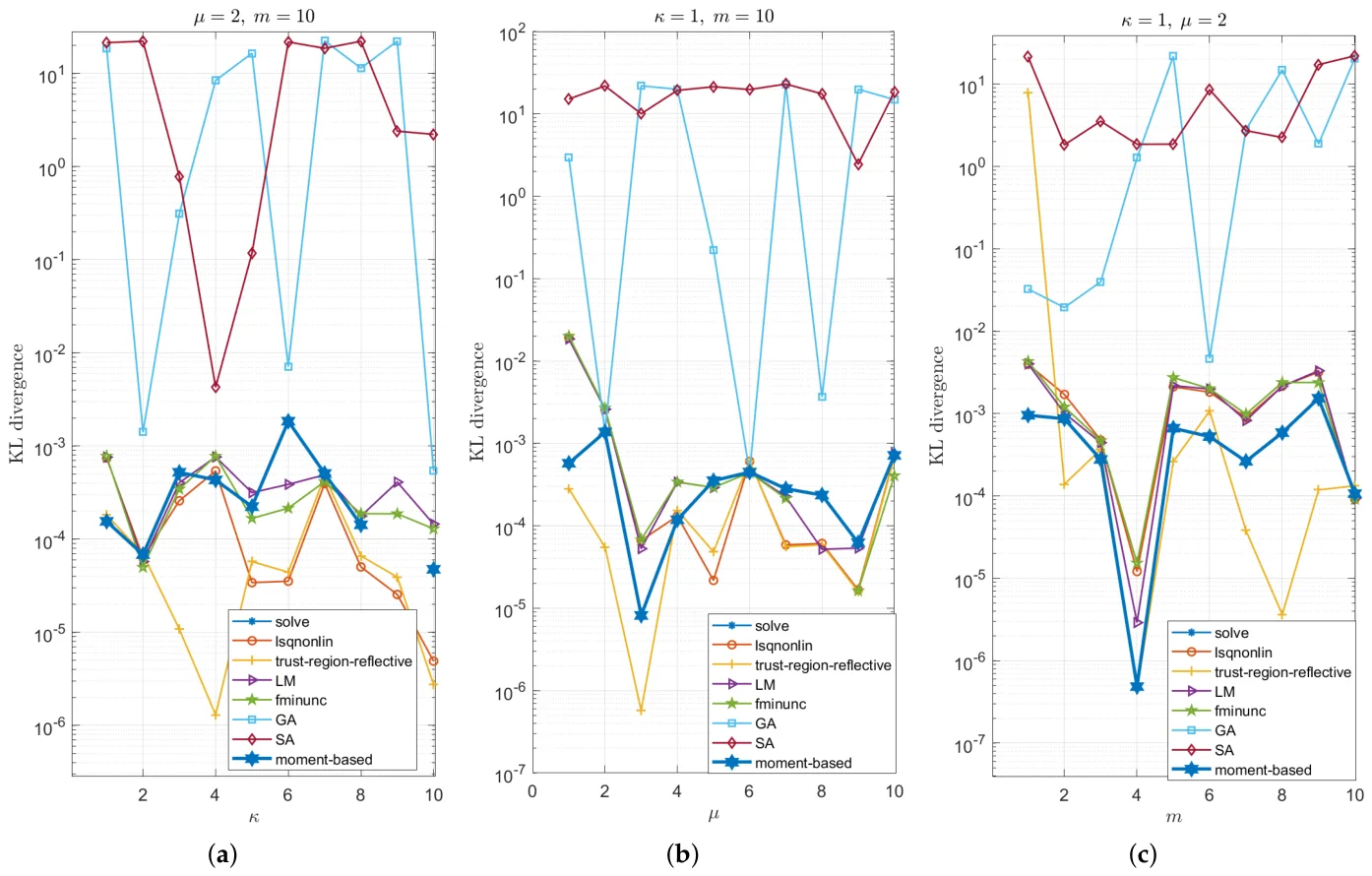
The KL divergence performances of the algorithms under different values of (κ, μ, *m*). (**a**) KL divergence for different κ values. (**b**) KL divergence for different μ values. (**c**) KL divergence for different *m* values.

Figure 3 further evaluates the distribution-reconstruction performance using the KL divergence between the true and estimated κ-μ shadowed distributions. The proposed moment-based estimator generally maintains a low KL divergence over the considered parameter ranges, indicating that the reconstructed distributions closely preserve the statistical characteristics of the target fading model. Together with the small PDF RMSE values reported in Table 3, these results indicate that deviations in the individually recovered parameters do not necessarily lead to substantial distributional discrepancies. This behavior is consistent with the nonlinear parameter coupling discussed in Section 3, whereby different combinations of κ, μ, and *m* can produce similar distribution shapes. In contrast, some conventional numerical solvers exhibit pronounced error peaks or fail to yield valid estimates for particular parameter settings, reflecting their sensitivity to the nonlinear and ill-conditioned parameter space. When an estimation method does not yield a physically admissible parameter estimate for a particular parameter setting, no reconstructed PDF is available for KL-divergence evaluation, and the corresponding KL result is recorded as NaN. These realization-level NaNs are distinct from numerically underflowed density values within the KL integration grid, for which the complete grid is retained using the numerical-floor and renormalization procedure described above. Consequently, the corresponding curve is interrupted rather than being connected across the missing KL result. Overall, the KL-divergence results indicate that, for realizations yielding admissible estimates, the proposed estimator provides high-fidelity distribution reconstruction without iterative numerical optimization.

### 4.4. Sensitivity to the Prescribed Composite Parameter *z*

Since the proposed closed-form estimator treats z=κμ/m as a pre-calibrated composite parameter, the effect of mismatch in the prescribed value of *z* is further investigated. Specifically, the value supplied to the estimator is defined as

(37) $$z_{\mathrm{used}}=z_{\mathrm{true}}\left(1+\delta_{z}\right),$$

where $\delta_{z}$ denotes the relative mismatch. In this experiment, $z_{\mathrm{true}}=0.2$, and $\delta_{z}$ is varied from −20% to 20%, corresponding to $z_{\mathrm{used}}\in \left[0.16,0.24\right]$.

**Figure 4:**
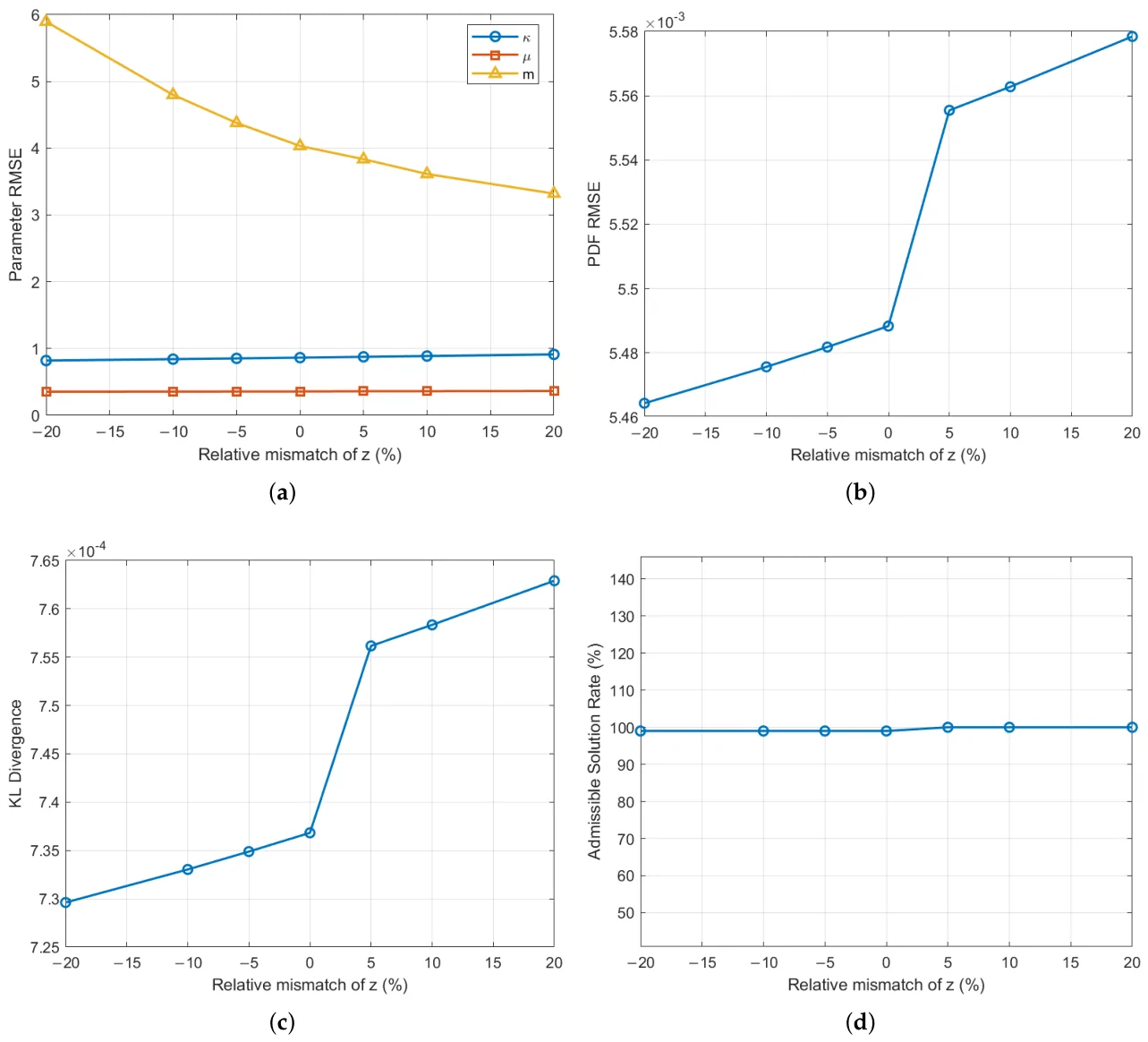
Sensitivity of the proposed estimator to mismatch in the prescribed composite parameter *z*. (**a**) RMSEs of the estimated κ, μ, and *m*. (**b**) PDF fitting RMSE. (**c**) KL divergence. (**d**) Admissible solution rate.

Figure 4 shows the RMSEs of the estimated parameters, the PDF fitting RMSE, the KL divergence, and the admissible solution rate under different levels of *z* mismatch. The individual parameters exhibit different sensitivities to the prescribed value of *z*. As $\delta_{z}$ increases from −20% to 20%, the RMSE of κ changes from 0.8201 to 0.9125, while that of μ changes only slightly from 0.3536 to 0.3636. In contrast, the RMSE of *m* changes substantially from 5.8945 to 3.3201, indicating that the recovery of *m* is considerably more sensitive to the prescribed value of *z*. This asymmetric behavior is associated with the coupling among κ, μ, and *m* through the composite relation z=κμ/m.

It is worth noting that the variation in the individual parameter RMSEs does not lead to a comparable deterioration in the reconstructed fading distribution. Over the entire mismatch range, the PDF RMSE varies only from $5.4643\times 10^{-3}$ to $5.5784\times 10^{-3}$, while the KL divergence remains between $7.2961\times 10^{-4}$ and $7.6291\times 10^{-4}$. Therefore, although the individual parameter estimates, particularly *m*, can be sensitive to the prescribed value of *z*, the resulting PDF fitting performance remains relatively robust to moderate *z* mismatch. Here, an admissible realization refers to a realization for which a unique estimate remains after applying the physical admissibility conditions and, only for parameter settings within the calibrated range when necessary, the calibrated parameter-domain criterion. For the present m=10 out-of-domain experiment, the domain criterion is not applied; realizations with two physically admissible roots are classified as unresolved. Infeasible or unresolved realizations are not counted as admissible.

Furthermore, the admissible solution rate remains between 99% and 100% for all investigated mismatch levels, demonstrating that the proposed admissibility criteria maintain high numerical feasibility over the considered range. These results indicate that accurate pre-calibration of *z* is important when the objective is accurate recovery of the individual model parameters, especially *m*, whereas moderate mismatch in *z* has a relatively limited effect on the reconstructed fading distribution.

### 4.5. Sample-Size Sensitivity

To further investigate the finite-sample behavior of the proposed estimator and assess the effect of the sample size adopted in the preceding simulations, an additional sample-size sensitivity experiment is conducted. The true channel parameters are fixed as κ=1, μ=2, and m=10, corresponding to z=0.2, while the sample size is varied as N∈ {100, 200, 500, 1000, 2000, 5000, 10,000}. For each sample size, 100 independent Monte Carlo trials are performed. The parameter RMSE, PDF fitting RMSE, KL divergence, and admissible solution rate are evaluated.

**Figure 5:**
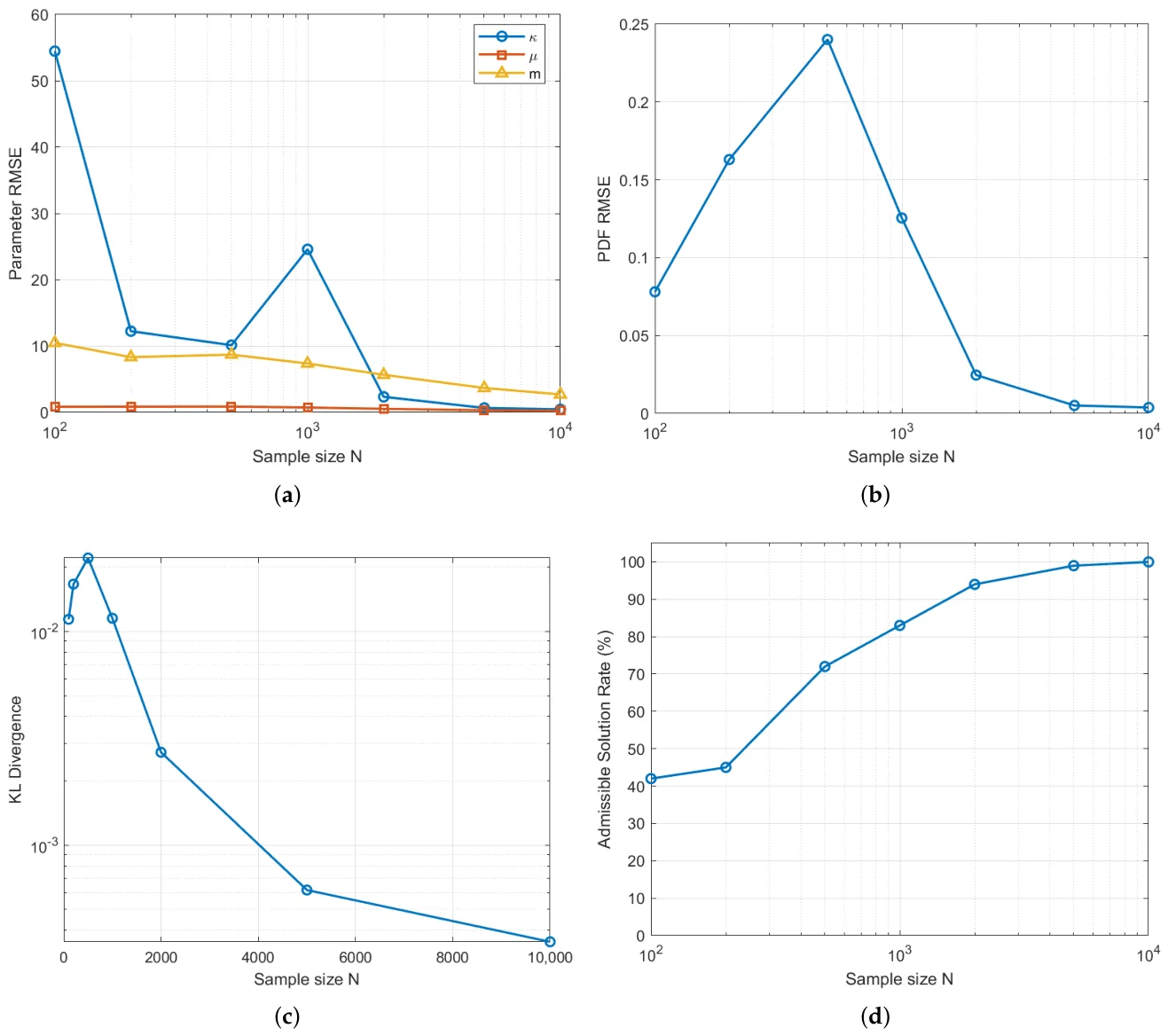
Sensitivity of the proposed estimator to the sample size *N*. (**a**) RMSEs of the estimated κ, μ, and *m*. (**b**) PDF fitting RMSE. (**c**) KL divergence. (**d**) Admissible solution rate.

Figure 5 illustrates the finite-sample behavior of the proposed estimator. As shown in Figure 5a, the parameter-estimation errors exhibit relatively large fluctuations for small sample sizes. This behavior is particularly evident for κ, whose estimate is sensitive to perturbations in the low-order sample moments when only a limited number of observations are available. As *N* increases, however, the parameter RMSEs generally decrease and become substantially more stable. In particular, when *N* increases from 2000 to 10,000, the RMSE of κ decreases from 2.3359 to 0.4538, while that of μ decreases from 0.5209 to 0.2312. The RMSE of *m* also decreases from 5.6617 to 2.6969.

More importantly, the distribution-level estimation accuracy improves markedly as the sample size becomes sufficiently large. As shown in Figure 5b,c, although the PDF RMSE and KL divergence exhibit some non-monotonic fluctuations in the small-sample regime, both metrics decrease substantially for larger *N*. At N=5000, the PDF RMSE and KL divergence are $5.15\times 10^{-3}$ and $6.17\times 10^{-4}$, respectively, and they further decrease to $3.83\times 10^{-3}$ and $3.53\times 10^{-4}$ at N= 10,000. These results indicate that the reconstructed fading distribution becomes increasingly stable as the accuracy of the estimated low-order moments improves with increasing sample size.

For small sample sizes, the accuracy of the sample moments is limited, and a considerable fraction of the Monte Carlo realizations may not satisfy the physical admissibility conditions. The PDF RMSE and KL divergence are therefore evaluated over the realizations yielding physically admissible parameter estimates. As the sample size increases, the sample moments become more stable, leading to a higher admissible solution rate and progressively improved distribution-reconstruction accuracy. In the sufficiently large-sample regime, both the PDF RMSE and KL divergence decrease markedly, demonstrating the improved statistical reliability of the proposed estimator.

The improvement in numerical feasibility with increasing sample size is more clearly reflected by the admissible solution rate in Figure 5d. The admissible rate increases from 42% at N=100 to 72% at N=500 and 83% at N=1000. It further reaches 94% at N=2000, 99% at N=5000, and 100% at N= 10,000. This trend indicates that increasing the number of observations reduces the fluctuations of the sample moments and substantially increases the probability that the closed-form solution satisfies the physical admissibility conditions.

Overall, the sample-size sensitivity results show that the proposed estimator becomes considerably more reliable when a sufficient number of observations is available. The choice of N=5000 in the preceding simulations provides an admissible solution rate of 99%, together with a PDF RMSE of approximately $5.15\times 10^{-3}$ and a KL divergence of approximately $6.17\times 10^{-4}$. Increasing the sample size to N= 10,000 provides further improvement, but the distribution-level gain is comparatively moderate. Therefore, N=5000 is retained in the preceding experiments as a practical operating point for evaluating the proposed estimator. It should be emphasized that N=5000 is used here as a representative simulation operating point rather than as a universal requirement for practical implementation. In rapidly time-varying channels where fewer approximately stationary observations are available within an estimation interval, the reduced admissible-solution rate and increased estimation variability observed for small *N* in Figure 5 should be taken into account.

For the fixed representative configuration κ=1, μ=2, and m=10, the finite-sample bias and variability of the parameter estimates were also examined. These quantities are distinct from the range-averaged biases reported in Figure 2, which summarize the overall estimation tendency across the randomly generated parameter configurations. At N=5000, the estimation biases of κ, μ, and *m* are 0.2224, −0.0860, and 0.7580, respectively, while the corresponding standard deviations are 0.6254, 0.3077, and 3.6087. At N= 10,000, the biases further decrease to 0.0997, −0.0406, and 0.2767, respectively, with standard deviations of 0.4449, 0.2288, and 2.6961. These results indicate that both the finite-sample bias and estimation variability are reduced as the number of observations increases, although the shadowing parameter *m* remains more difficult to recover accurately than κ and μ.

These empirical observations motivate a further analytical characterization of the estimator bias and variance and their comparison with the corresponding constrained Cramér–Rao lower bound, as presented in the following subsection.

### 4.6. Asymptotic Statistical Analysis and Constrained CRLB

To complement the finite-sample Monte Carlo analysis in Section 4.5, the asymptotic statistical behavior of the proposed estimator is further investigated. Let $M_{j}=E\left[\rho^{j}\right]$ denote the *j*-th population raw moment and define the sample-moment vector as $U={\left[U_{1},U_{2},U_{3}\right]}^{T}$, where $U_{j}=N^{-1}\sum_{n=1}^{N}\rho_{n}^{j}$. Under independent sampling and assuming that the sixth-order moment is finite, the multivariate central limit theorem gives [18]

(38) $$\sqrt{N}\left(U-M\right)\overset{d}{\to }N\left(0,\Omega_{U}\right),$$

where $M={\left[M_{1},M_{2},M_{3}\right]}^{T}$ and

(39) $${\left[\Omega_{U}\right]}_{ij}=M_{i+j}-M_{i}M_{j},\;i,j=1,2,3.$$

Accordingly, $\mathrm{Cov}\left(U\right)\approx \Omega_{U}/N$. Although only the first three moments are required for the proposed parameter estimator, moments up to the sixth order are used here solely to characterize its asymptotic covariance.

The normalized moments used for parameter estimation are $\hat{q}={\left[{\hat{Q}}_{2},{\hat{Q}}_{3}\right]}^{T}$, where ${\hat{Q}}_{2}=U_{2}/U_{1}^{2}$ and ${\hat{Q}}_{3}=U_{3}/U_{1}^{3}$. Defining $\hat{q}=g\left(U\right)$, the Jacobian matrix of this transformation evaluated at the population moments is

(40) $$G=\left[\begin{array}{ccc} -2M_{2}/M_{1}^{3} & 1/M_{1}^{2} & 0\\ -3M_{3}/M_{1}^{4} & 0 & 1/M_{1}^{3} \end{array}\right].$$

Application of the multivariate delta method [18] therefore yields

(41) $$\sqrt{N}\left(\hat{q}-q\right)\overset{d}{\to }N\left(0,\Omega_{q}\right),\;\Omega_{q}=G\Omega_{U}G^{T},$$

and hence

(42) $$\mathrm{Cov}\left(\hat{q}\right)\approx \frac{1}{N}\Omega_{q}.$$

For a prescribed value of *z*, the recovered parameter vector can be expressed as $\hat{\theta }=h\left(\hat{q};z\right)$, where $\theta ={\left[\kappa ,\mu ,m\right]}^{T}$. Applying the delta method again gives

(43) $$\Sigma_{\theta }≜\mathrm{Cov}\left(\hat{\theta }\right)\approx \frac{1}{N}H\Omega_{q}H^{T},$$

where

(44) $$H=\frac{\partial (\kappa ,\mu ,m)}{\partial (Q_{2},Q_{3})}.$$

The required derivatives can be obtained from the closed-form estimator. From

(45) $$F\left(P,Q_{2},Q_{3}\right)=DP^{2}-2\left(z+2\right)\left(Q_{2}-1\right)P+2\left(z+1\right)=0,$$

where $D=Q_{3}-3Q_{2}+2$, implicit differentiation gives

(46) $$\frac{\partial P}{\partial Q_{2}}=\frac{3P^{2}+2\left(z+2\right)P}{2DP-2\left(z+2\right)\left(Q_{2}-1\right)},\;\frac{\partial P}{\partial Q_{3}}=-\frac{P^{2}}{2DP-2\left(z+2\right)\left(Q_{2}-1\right)}.$$

Using $d=[\left(Q_{2}-1\right)P^{2}-\left(z+1\right)P]/z$ and defining $d_{P}=\left[2\left(Q_{2}-1\right)P-\left(z+1\right)\right]/z$, the corresponding derivatives are

(47) $$\frac{\partial d}{\partial Q_{2}}=\frac{P^{2}}{z}+d_{P}\frac{\partial P}{\partial Q_{2}},\;\frac{\partial d}{\partial Q_{3}}=d_{P}\frac{\partial P}{\partial Q_{3}}.$$

The derivatives of the original parameters then follow from μ=(P−zd)/(z+1), m=d+μ, and κ=zm/μ as

(48) $$\frac{\partial \mu }{\partial Q_{i}}=\frac{\partial P/\partial Q_{i}-z\;\partial d/\partial Q_{i}}{z+1},\;\frac{\partial m}{\partial Q_{i}}=\frac{\partial d}{\partial Q_{i}}+\frac{\partial \mu }{\partial Q_{i}},\;i=2,3,$$

and

(49) $$\frac{\partial \kappa }{\partial Q_{i}}=\frac{z}{\mu^{2}}\left(\mu \frac{\partial m}{\partial Q_{i}}-m\frac{\partial \mu }{\partial Q_{i}}\right),\;i=2,3.$$

Therefore, the parameter variances decrease asymptotically as $O(N^{-1})$, while the corresponding standard deviations decrease as $O(N^{-1/2})$.

Because ${\hat{Q}}_{2}$ and ${\hat{Q}}_{3}$ are nonlinear ratios of sample moments, they are generally biased for finite *N*. Using a second-order delta expansion [19], their leading-order biases can be approximated by

(50) $$\mathrm{Bias}\left({\hat{Q}}_{r}\right)\approx \frac{1}{2N}\mathrm{tr}\left(H_{g_{r}}\Omega_{U}\right),\;r=2,3,$$

where $H_{g_{r}}$ denotes the Hessian matrix of $g_{r}\left(U\right)$. Propagating these terms through the parameter transformation gives

(51) $$\mathrm{Bias}\left({\hat{\theta }}_{l}\right)\approx \nabla h_{l}^{T}\mathrm{Bias}\left(\hat{q}\right)+\frac{1}{2N}\mathrm{tr}\left(H_{h_{l}}\Omega_{q}\right),$$

where $\theta_{l}\in \left\{\kappa ,\mu ,m\right\}$ and $H_{h_{l}}$ is the Hessian matrix of the corresponding parameter transformation. Thus, the leading finite-sample bias is of order $O(N^{-1})$ and gradually vanishes as the sample size increases. This result does not imply exact finite-sample unbiasedness; rather, the estimator approaches the corresponding population-moment solution under the prescribed-*z* constraint as *N* increases.

Based on the asymptotic covariance matrix in (43), an approximate 95% confidence interval for the *l*-th recovered parameter can be written as

(52) $${\hat{\theta }}_{l}\pm 1.96\sqrt{{\left[\Sigma_{\theta }\right]}_{ll}},$$

where $\theta_{l}\in \left\{\kappa ,\mu ,m\right\}$. This interval provides an asymptotic measure of the uncertainty associated with the recovered parameters under the prescribed-*z* constraint.

To further quantify the statistical efficiency of the proposed estimator, the Fisher information matrix (FIM) is considered. For one observation, with $\theta ={\left[\kappa ,\mu ,m\right]}^{T}$, the FIM is defined as [20]

(53) $${\left[I\left(\theta \right)\right]}_{ij}=E\;\left[\frac{\partial \ln f_{\rho }}{\partial \theta_{i}}\frac{\partial \ln f_{\rho }}{\partial \theta_{j}}\right]=\int_{0}^{\infty }\frac{\partial \ln f_{\rho }}{\partial \theta_{i}}\frac{\partial \ln f_{\rho }}{\partial \theta_{j}}f_{\rho }\left(\rho ;\theta \right)\;d\rho .$$

Since the proposed estimator uses the prescribed composite parameter z=κμ/m, the parameters satisfy the equality constraint c(θ)=κμ/m−z=0, whose gradient is $C=\partial c\left(\theta \right)/\partial \theta^{T}=\left[\mu /m,\;\kappa /m,\;-\kappa \mu /m^{2}\right]$. Accordingly, rather than using the unconstrained Cramér–Rao lower bound, the constrained Cramér–Rao bound (CCRB) under the same equality constraint is adopted as the statistical-efficiency reference [21,22]. For *N* independent observations,

(54) $$B_{\mathrm{CCRB}}=\frac{1}{N}\left[I^{-1}-I^{-1}C^{T}{\left(CI^{-1}C^{T}\right)}^{-1}CI^{-1}\right].$$

In the numerical evaluation, the score derivatives $\partial \ln f_{\rho }/\partial \theta_{i}$ are evaluated at the specified parameter point, and the expectations in (53) are computed by numerical quadrature over the positive SNR support. The resulting Fisher information matrix is then substituted into (54), together with the analytical constraint gradient *C*, to obtain the CCRB. The reported standard-deviation bounds are calculated as $\sigma_{\mathrm{CCRB},i}=\sqrt{{\left[B_{\mathrm{CCRB}}\right]}_{ii}}$. The diagonal entries of $B_{\mathrm{CCRB}}$ provide the corresponding variance-bound references for κ, μ, and *m*.

To numerically verify the above analysis, the fixed representative parameter setting κ=1, μ=2, m=10, and z=0.2 is considered with N=5000. A total of 1000 independent Monte Carlo realizations are used for the empirical bias and variance evaluation. Therefore, the bias values reported here characterize this specific parameter configuration and should not be directly compared with the range-averaged biases reported in Figure 2. The analytical and empirical results are summarized in Table 4.

Here, $b_{\mathrm{MC}}$ and $b_{A}$ denote the empirical Monte Carlo bias and the second-order analytical bias, respectively, while $\sigma_{\mathrm{MC}}$, $\sigma_{A}$, and $\sigma_{\mathrm{CCRB}}$ denote the empirical standard deviation, the delta-method asymptotic standard deviation, and the constrained Cramér–Rao standard deviation bound, respectively. As shown in Table 4, the analytical bias approximation correctly predicts the signs and the orders of magnitude of the empirical biases for all three parameters. At N=5000, the empirical biases of κ, μ, and *m* are 0.2127, −0.0761, and 0.6196, respectively, whereas the corresponding analytical approximations are 0.1531, −0.0633, and 0.4104. The remaining differences are expected because the second-order expansion retains only the leading terms of the finite-sample bias.

The empirical and asymptotic standard deviations also show good agreement for μ and *m*. Specifically, the empirical and analytical standard deviations are 0.3068 and 0.3108 for μ, and 3.5888 and 3.7246 for *m*, respectively. A larger difference is observed for κ, for which the empirical and analytical standard deviations are 0.7223 and 0.5260. This discrepancy can be attributed to the stronger local sensitivity of κ to perturbations in the normalized moments and to the nonlinear transformation from $\left(Q_{2},Q_{3}\right)$ to the original channel parameters. Nevertheless, the asymptotic analysis correctly captures the overall magnitude and sample-size dependence of the estimation variability.

The CCRB standard deviations for κ, μ, and *m* are 0.2348, 0.1291, and 1.7388, respectively. Defining the constrained asymptotic efficiency as

(55) $$\eta_{i}=\frac{{\left[B_{\mathrm{CCRB}}\right]}_{ii}}{{\left[\Sigma_{\theta }\right]}_{ii}},$$

the corresponding efficiencies are approximately 0.1992, 0.1726, and 0.2180 for κ, μ, and *m*, respectively. Therefore, the proposed closed-form moment estimator does not attain the CCRB. Instead, it trades part of the statistical efficiency for a substantial reduction in computational complexity by avoiding iterative numerical optimization. This behavior is consistent with the main objective of the proposed method, namely, to provide a computationally efficient estimator with accurate distribution reconstruction rather than to construct a minimum-variance parameter estimator.

It is also worth noting that the Fisher information matrix at the representative parameter point is strongly ill-conditioned, with a reciprocal condition number of approximately $6.17\times 10^{-8}$. This observation provides additional quantitative evidence of the strong coupling and identifiability difficulty among κ, μ, and *m*. In particular, the relatively weak information associated with the shadowing parameter is consistent with the larger uncertainty observed in the estimation of *m*. These results also help explain why noticeably different parameter combinations can still generate similar fading distributions, as observed in Section 4.3.

Because the normalized-moment estimator is biased for finite *N*, the CCRB is interpreted here as an asymptotic efficiency reference rather than as a strict finite-sample lower bound. Together with the sample-size results in Section 4.5, the analytical and Monte Carlo results show that both the bias and estimation variability decrease as *N* increases, while the proposed estimator retains its closed-form and low-complexity characteristics.

### 4.7. Communication-Performance Evaluation

The preceding results demonstrate that the proposed estimator provides robust distribution reconstruction under both individual parameter discrepancies and moderate mismatch in the prescribed composite parameter *z*. To further assess whether this distribution-level robustness translates into reliable communication-performance prediction, three representative performance metrics are considered: ergodic capacity [23], symbol error rate (SER) [24], and outage probability (OP). These metrics provide complementary assessments of the reconstructed channel model because they exhibit different sensitivities to the shape and local characteristics of the SNR distribution.

To ensure reproducibility of the performance-level evaluation, the three metrics are calculated from the true and reconstructed κ-μ shadowed SNR distributions. The ergodic capacity is evaluated as

(56) $$C=\int_{0}^{\infty }\log_{2}\left(1+\gamma \right)f_{\gamma }\left(\gamma \right)\;d\gamma .$$

For the SER-based performance evaluation, coherent binary phase-shift keying (BPSK) signaling is adopted as a representative modulation format. It should be emphasized that the proposed parameter-estimation procedure itself is modulation-independent and operates directly on instantaneous-SNR samples; BPSK is introduced only for evaluating the communication performance associated with the reconstructed fading distributions. For M-ary phase-shift keying (*M*-PSK) signaling, the average SER is evaluated using the MGF-based expression

(57) $$P_{\mathrm{SER}}=\frac{1}{\pi }\int_{0}^{(M-1)\pi /M}M_{\gamma }\left(-\frac{\sin^{2}\left(\pi /M\right)}{\sin^{2}\theta }\right)d\theta ,$$

where $M_{\gamma }\left(\cdot \right)$ denotes the MGF of the instantaneous SNR given in (2). In this work, M=2 is adopted, corresponding to coherent BPSK.

The outage probability is calculated as

(58) $$P_{\mathrm{out}}=\mathrm{Pr}\left(\gamma <\gamma_{\mathrm{th}}\right)=\int_{0}^{\gamma_{\mathrm{th}}}f_{\gamma }\left(\gamma \right)\;d\gamma ,$$

where the outage threshold is fixed at $\gamma_{\mathrm{th}}=0.5$ in linear scale for all numerical evaluations.

**Figure 6:**
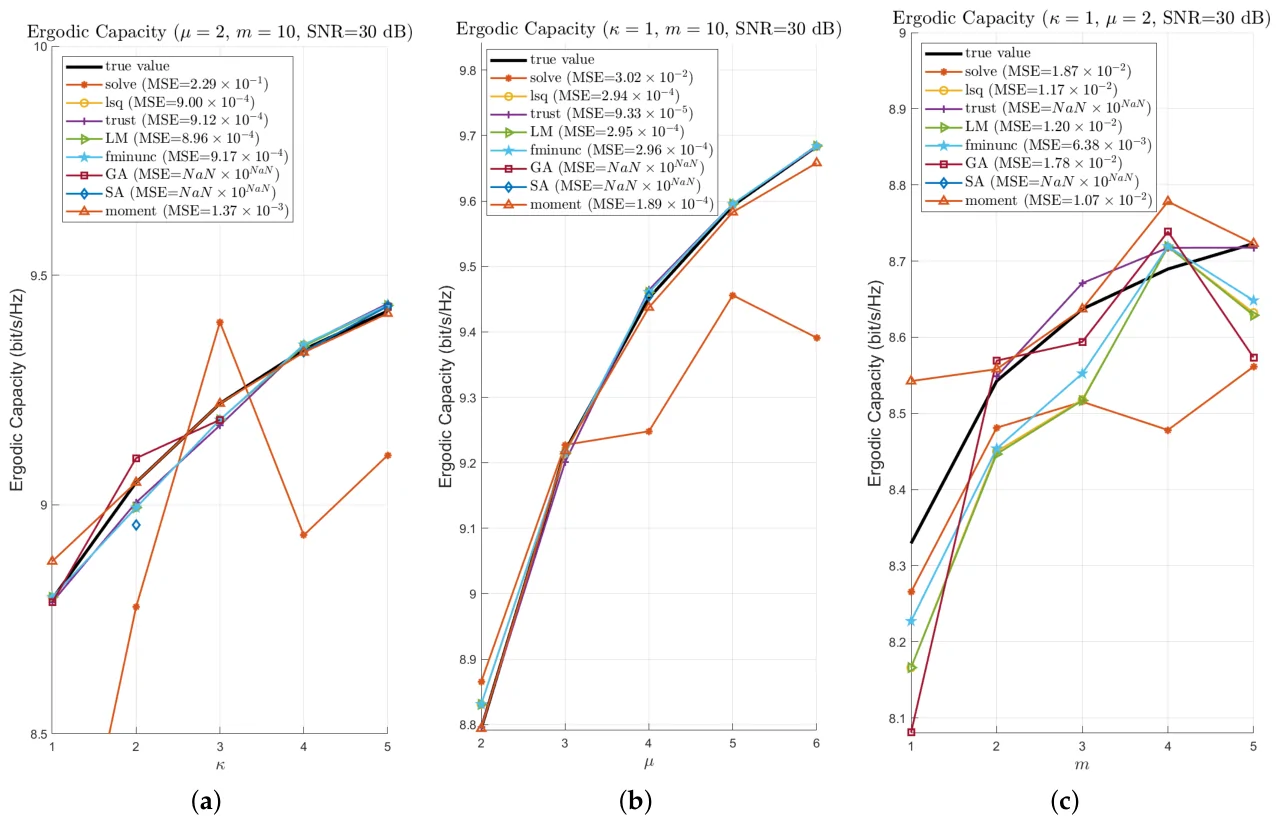
Comparison of ergodic capacity under different estimation algorithms. (**a**) Ergodic capacity for different κ values. (**b**) Ergodic capacity for different μ values. (**c**) Ergodic capacity for different *m* values.

Figure 6 compares the ergodic capacity obtained using the parameters recovered by different estimation methods with that calculated from the true channel parameters. The proposed moment-based estimator closely follows the true ergodic-capacity curves over most of the considered κ, μ, and *m* ranges and achieves a small capacity-prediction mean square error (MSE). This result is particularly relevant when considered together with Figure 2 and Table 3. Although the individual recovered parameters may differ from their generating values, the resulting distribution can still accurately reproduce the ergodic capacity. Therefore, the residual parameter discrepancies do not necessarily translate into comparable errors in link-quality prediction. This result indicates that preserving the relevant statistical characteristics of the fading distribution can support accurate capacity evaluation, demonstrating the potential of the proposed estimator for link-quality assessment in WSNs.

**Figure 7:**
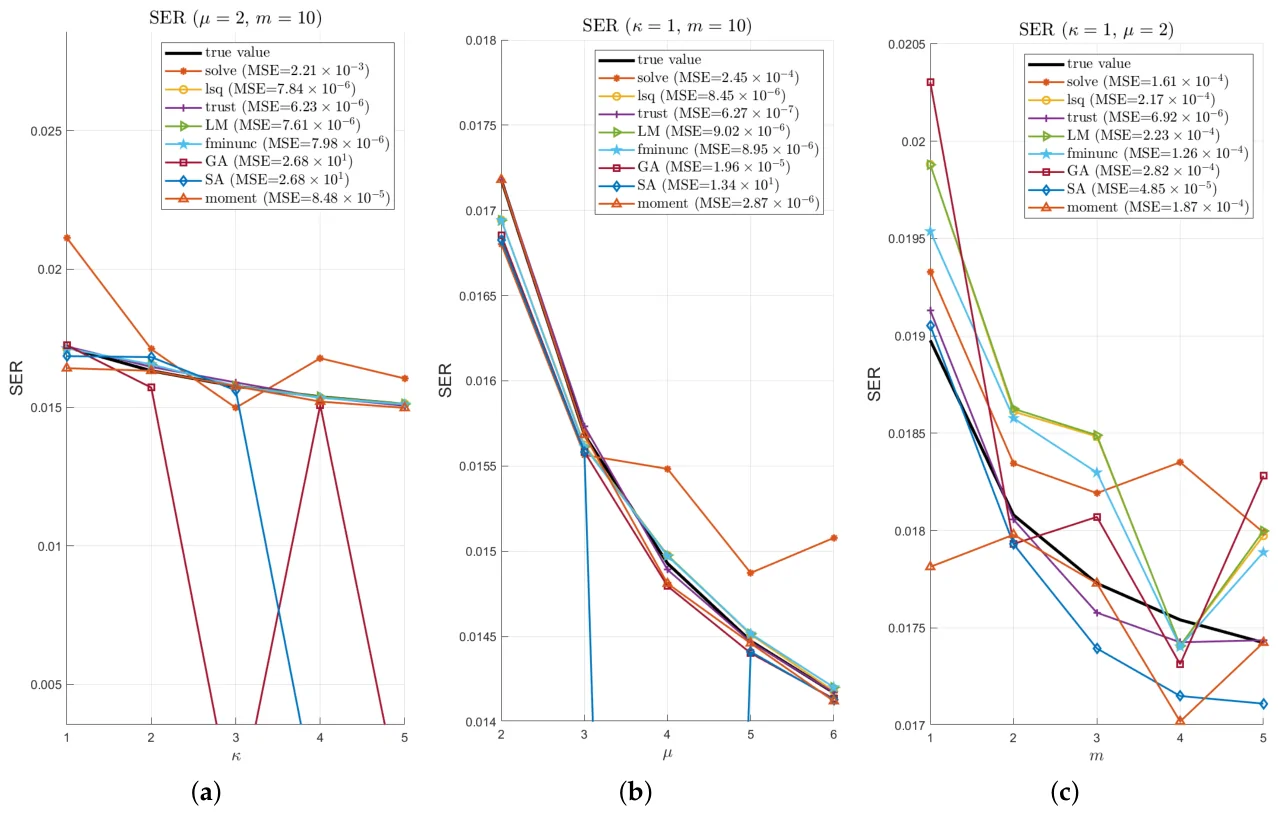
Comparison of SER under different estimation algorithms. (**a**) SER for different κ values. (**b**) SER for different μ values. (**c**) SER for different *m* values.

Figure 7 presents the corresponding SER results. Compared with the ergodic-capacity prediction, relatively larger discrepancies are observed for some parameter configurations. This difference arises because SER is more sensitive to particular regions of the instantaneous-SNR distribution than ergodic capacity. The proposed estimator is constructed from the low-order normalized moments $Q_{2}$ and $Q_{3}$, which effectively preserve the global statistical characteristics of the fading distribution but do not explicitly constrain its local or tail behavior. Consequently, even a small PDF fitting error may lead to a more noticeable SER deviation at a given operating SNR. Nevertheless, the proposed estimator generally captures the variation trend of SER as the channel parameters change.

**Figure 8:**
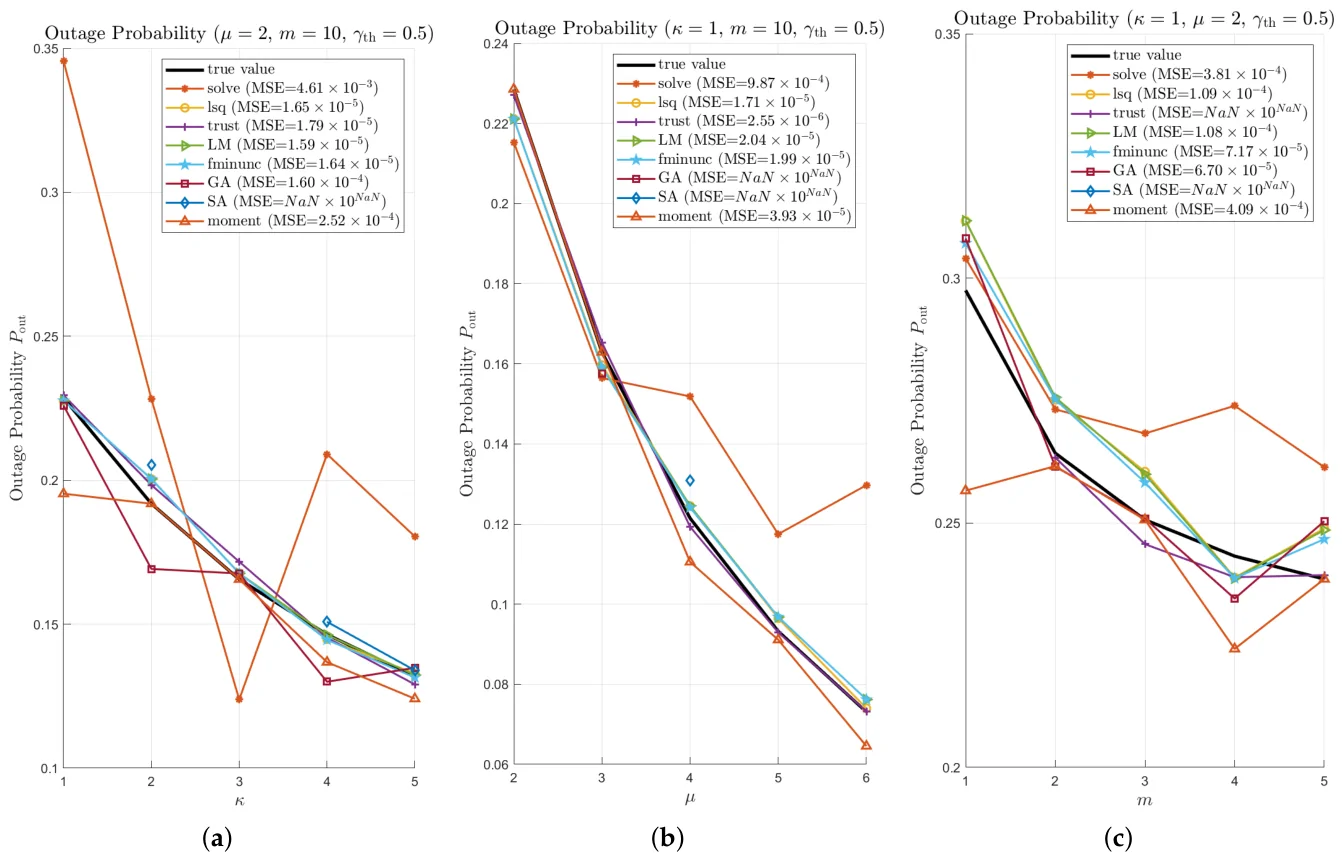
Comparison of outage probability under different estimation algorithms. (**a**) Outage probability for different κ values. (**b**) Outage probability for different μ values. (**c**) Outage probability for different *m* values.

A similar behavior is observed for the outage probability in Figure 8. Because outage probability is determined by the cumulative probability below a prescribed SNR threshold, it is particularly sensitive to discrepancies in the lower-SNR region of the reconstructed distribution. Therefore, a small global PDF RMSE does not necessarily guarantee an equally small outage-probability error. The deviations observed for some parameter settings reflect this tail sensitivity rather than a failure to reconstruct the overall fading distribution. Despite these local discrepancies, the proposed estimator reproduces the general dependence of outage probability on the channel parameters while retaining its low computational complexity.

## 5. Conclusions

This paper has addressed the problem of computationally efficient parameter estimation for the κ-μ shadowed fading model in WSNs. By exploiting the algebraic structure of the normalized moment equations, a reparameterization framework was developed to reduce the coupled nonlinear estimation problem to a closed-form quadratic solution under a prescribed composite parameter *z*. The proposed estimator avoids iterative numerical optimization in its core estimation stage while providing competitive distribution-reconstruction accuracy. The simulation results show that, although deviations may remain in the recovered individual parameters, the reconstructed fading distributions generally preserve the relevant statistical characteristics, as demonstrated by the PDF RMSE and KL divergence. The sample-size and *z*-mismatch analyses further characterize the finite-sample behavior and robustness of the proposed framework. In addition, the reconstructed distributions provide accurate ergodic-capacity prediction over most of the considered parameter ranges, indicating the potential usefulness of the proposed estimator for link-quality and communication-performance evaluation. Nevertheless, a limitation of the present study is that the validation is based on synthetically generated κ-μ shadowed samples rather than measured CMC or vehicular channel data. Moreover, the proposed framework assumes that the composite parameter *z* is available from prior calibration. Future work will therefore investigate the environment-dependent stability and data-driven calibration of *z* using measured channel data, as well as the integration and validation of the proposed estimator in practical adaptive communication systems.

## Figures and Tables

**Table 3:** True values and RMSE of κ, μ, *m*.

$\kappa_{\mathrm{real}}$	$\mu_{\mathrm{real}}$	$m_{\mathrm{real}}$	$\kappa_{\mathrm{RMSE}}$	$\mu_{\mathrm{RMSE}}$	$m_{\mathrm{RMSE}}$	${\mathrm{PDF}}_{\mathrm{RMSE}}$
1.423	2.701	4.538	0.388313	0.251962	0.849183	0.004639
0.644	4.052	5.398	0.431382	0.403581	2.626620	0.005482
0.531	3.035	4.898	0.269289	0.174312	2.114849	0.004019
1.147	1.709	4.444	0.608033	0.231501	1.247786	0.007937
1.467	2.893	4.890	0.574239	0.361248	1.054579	0.005120
0.904	4.083	3.292	0.237863	0.226558	0.638909	0.004118
1.963	2.139	3.447	0.532453	0.262458	0.561954	0.006377
1.401	2.114	2.913	0.518150	0.252475	0.651524	0.007415
1.014	3.360	3.175	0.353744	0.261443	0.706446	0.004925
0.845	3.361	4.524	0.365601	0.292367	1.581848	0.005529

**Table 4:** Asymptotic statistical performance and constrained Cramér–Rao bound (CCRB) for κ=1, μ=2, m=10, z=0.2, and N=5000.

Parameter	$b_{\mathrm{MC}}$	$b_{A}$	$\sigma_{\mathrm{MC}}$	$\sigma_{A}$	$\sigma_{\mathrm{CCRB}}$
κ	0.2127	0.1531	0.7223	0.5260	0.2348
μ	−0.0761	−0.0633	0.3068	0.3108	0.1291
*m*	0.6196	0.4104	3.5888	3.7246	1.7388

## Data Availability

The original contributions presented in this study are included in the article. Further inquiries can be directed to the corresponding author(s).

## References

[1] Rawat P., Singh K.D., Chaouchi H., Bonnin J.M. (2014). Wireless sensor networks: A survey on recent developments and potential applications. J. Supercomput..

[2] Sharma S.K., Chawla M. (2023). Compatibility analysis of cluster-based WSN framework for IoT applications. Wirel. Pers. Commun..

[3] Moreno-Pozas L., Lopez-Martinez F.J., Paris J.F., Martos-Naya E. (2016). The *κ*–*μ* shadowed fading model: Unifying the *κ*–*μ* and *η*–*μ* distributions. IEEE Trans. Veh. Technol..

[4] Al-Rimawi A., Al-Dweik A. (2022). On the Performance of RIS-Assisted Communications with Direct Link over *κ*-*μ* Shadowed Fading. IEEE Open J. Commun. Soc..

[5] Ding J., Xie X., Wang L., Tan L., Ma J., Kang D. (2022). Performance of Dual-Hop FSO/RF Systems with Fixed-Gain Relaying over Fisher–Snedecor *F* and *κ*-*μ* Shadowed Fading Channels. Appl. Opt..

[6] Le-Thanh T., Ho-Van K. (2023). Performance Analysis of Wireless Communications with Nonlinear Energy Harvesting under Hardware Impairment and *κ*-*μ* Shadowed Fading. Sensors.

[7] Chauhan P.S., Sau P.C., Kumar S., Jain A., Bhatia V. (2025). SEP Analysis on the *κ*-*μ* Shadowed Fading with Additive Laplacian Noise. Digit. Signal Process..

[8] Cañete F.J., López-Fernández J., García-Corrales C., Sánchez A., Robles E., Rodrigo F.J., Paris J.F. (2016). Measurement and Modeling of Narrowband Channels for Ultrasonic Underwater Communications. Sensors.

[9] Cotton S.L. (2016). Second-Order Statistics of *κ*-*μ* Shadowed Fading Channels. IEEE Trans. Veh. Technol..

[10] Vega Sánchez J.D., Urquiza-Aguiar L., Paredes Paredes M.C. (2021). Fading Channel Models for mm-Wave Communications. Electronics.

[11] Tian J., Fan Z., Ji Z., Li X., Fei P., Hou D. (2023). Multi-parameter estimation method and closed-form solution study for *κ*–*μ* channel model. Sensors.

[12] Xiang Y., Xie L., Li X., Du Q., Sun W., Huang Z., Hou D., Tian J. (2026). Suboptimal joint multiparameter estimation and performance analysis for wireless sensor networks over *κ*–*μ* fading channels. IEEE Sens. J..

[13] Asplund H., Molisch A.F., Steinbauer M., Mehta N.B. Clustering of scatterers in mobile radio channels-evaluation and modeling in the COST259 directional channel model. Proceedings of the IEEE International Conference on Communications.

[14] Paris J.F. (2014). Statistical characterization of *κ*–*μ* shadowed fading. IEEE Trans. Veh. Technol..

[15] Li Y.W., Qi F. (2024). A new closed-form formula of the Gauss hypergeometric function at specific arguments. Axioms.

[16] Martino L., Luengo D., Míguez J. (2018). Accept–reject methods. Independent Random Sampling Methods.

[17] Sen P.K., Singer J.M. (1993). Large Sample Methods in Statistics: An Introduction with Applications.

[18] van der Vaart A.W. (1998). Asymptotic Statistics.

[19] Oehlert G.W. (1992). A note on the delta method. Am. Stat..

[20] Kay S.M. (1993). Fundamentals of Statistical Signal Processing, Volume I: Estimation Theory.

[21] Gorman J.D., Hero A.O. (1990). Lower bounds for parametric estimation with constraints. IEEE Trans. Inf. Theory.

[22] Li X., Liu Y., Yang L. (2022). New alternative derivations of Cramér–Rao bound for equality-constrained estimation. Electron. Lett..

[23] García-Corrales C., Cañete F.J., París J.F. (2014). Capacity of *κ*–*μ* shadowed fading channels. Int. J. Antennas Propag..

[24] Ilic-Delibasic M., Pejanovic-Djurisic M. Symbol error rate for Ricean fading channel with random K parameter. Proceedings of the 14th International Symposium on Wireless Personal Multimedia Communications (WPMC).

